# Gastric duplication cyst in an adult with autoimmune hemolytic anemia: a case report and review of the literature

**DOI:** 10.1186/s13256-018-1895-5

**Published:** 2018-12-26

**Authors:** Amal Bennani, A. Miry, I. Kamaoui, T. Harroudi

**Affiliations:** 1 Department of Pathology, Mohamed I University, 30050 Oujda, Morocco; 2 Department of Radiology, Mohamed I University, 30050 Oujda, Morocco; 3 Department of Surgical Oncology, Mohamed I University, 30050 Oujda, Morocco; 4 Assaada, Bp 6210, 60020 Oujda, Morocco

**Keywords:** Duplication, Abdominal cyst, Gastric, Epithelium

## Abstract

**Background:**

Gastric duplication cysts are uncommon congenital anomalies found primarily in children and rarely seen in the adult population. Accurate diagnosis of cysts before resection is difficult even using the most advanced imaging techniques.

**Case presentation:**

In this report, we describe a 28-year-old Moroccan patient with a history of autoimmune hemolytic anemia who presented with an asymptomatic abdominal cystic mass detected during abdominal computed tomography performed before splenectomy. Magnetic resonance imaging performed for accurate characterization showed a high-signal-intensity cystic mass on T2-weighted images, located between the patient’s stomach and spleen. The patient underwent a complete cyst resection during exploratory laparotomy. The histological examination showed a cyst lined by three different epithelia with bundles of smooth muscle, which suggested a gastric duplication cyst.

**Conclusions:**

We report a case of gastric cyst duplication in an adult with autoimmune hemolytic anemia, and we discuss this rare association, radiological findings, and the unique histological findings of this case.

## Background

A gastrointestinal duplication is defined as a spherical cystic structure lined by a mucous membrane with smooth muscle in its wall [1]. It may occur anywhere along the mesenteric side of the gastrointestinal tract, most commonly in the ileum (35%). Gastric duplication cysts (GDCs) are a rare finding and account for only 2–9% of all gastrointestinal duplications [2]. Although most cases are diagnosed within the first year of life, a few cases have been diagnosed in adulthood [3]. The diagnosis of these cysts using all advanced imaging techniques before resection is difficult. We report a rare case of a patient with GDC with the aim of shedding more light on this uncommon developmental anomaly and on the fact that it should be considered in the differential diagnosis of abdominal cystic masses in adulthood, although the clinical and radiological findings are somewhat nonspecific. The current case is unique because it has an unusual histological finding and it is associated with an autoimmune hemolytic anemia, which allowed us to discuss a possible morbid association.

## Case presentation

We present a case of a 28-year-old white man with an 8-month history of clinical symptoms of anemia, such as fatigue and breathlessness, without any abdominal complaint. He had autoimmune hemolytic anemia treated by corticosteroids for more than 6 months but with a relapse of disease after steroid remission. For this reason, he was a candidate for a splenectomy. His occupation is a student. He did not smoke tobacco or consume alcohol, and he was taking prednisone, bisphosphonates, vitamin D, and calcium. No other immune disorders were found. On admission, his blood pressure was 90/70 mmhg, his heart rate was 70 beats/minute, and his body temperature was 36 °C. Physical examination of the patient revealed a severe pale conjunctiva and icteric sclera with no evidence of abdominal mass or other physical abnormalities. Laboratory analysis results on admission are shown in Table 1.

**Table 1:** Laboratory data on admission

Test	Results
Hematology	
WBC	11.5 × 10^9^/L
RBC	2.2 × 10^12^/L
Hb	6.8 g/L
Hct	24.3%
MCV	110 fl
Plt	400 × 10^9^/L
RC	18%
Biochemistry	
TP	80 g/L
CRP	4 mg/L
Urea	0.3 g/L
Creatinine	7 mg/L

*Abbreviations: Cr* Creatinine, *CRP* C-reactive protein, *Hb* Hemoglobin, *Hct* Hematocrit, *MCV* Mean corpuscular volume, *Plt* Platelets, *RBC* Red blood cells, *RC* Reticulocyte count, *TP* Total protein, *WBC* White blood cells

**Fig. 1:**
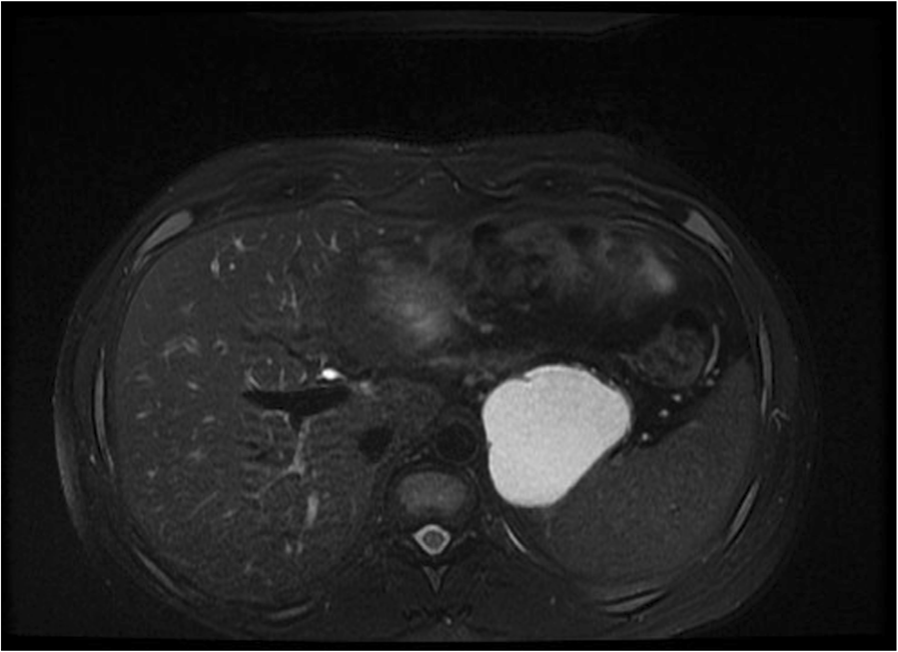
T2-weighted fat-saturated magnetic resonance image shows a high-signal-intensity cystic mass between stomach and spleen

**Fig. 2:**
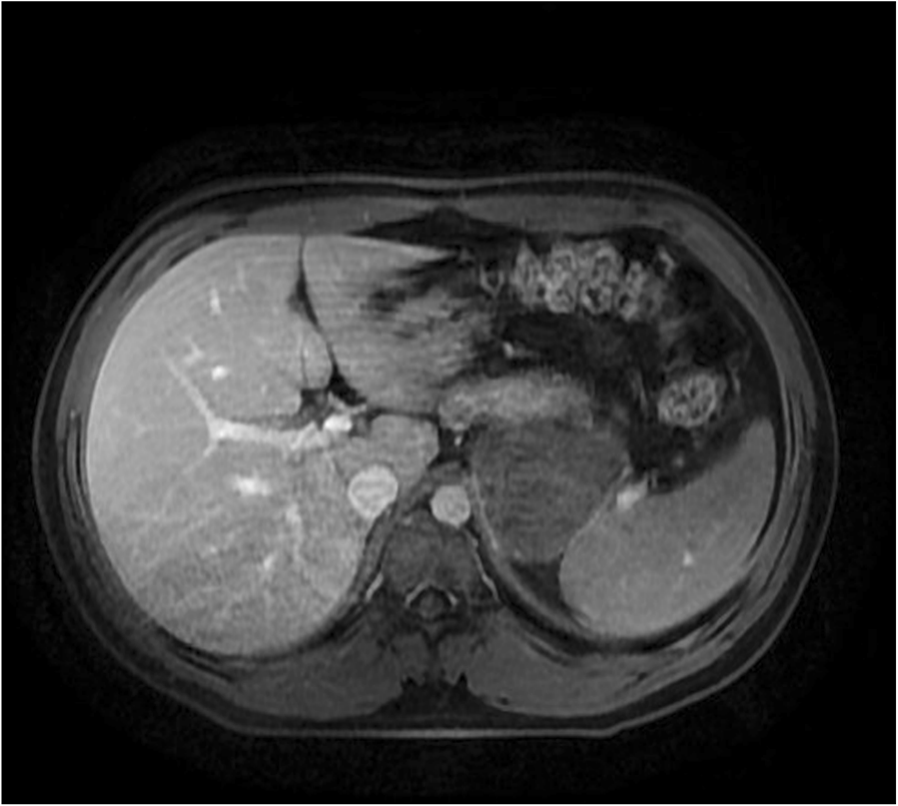
Axial T1-weighted gadolinium contrast-enhanced magnetic resonance image shows the larger cystic mass with peripheral enhancement

Abdominal computed tomography (CT) performed before the splenectomy showed a large cystic mass between the stomach and the spleen with no evidence of communication with the stomach or pancreas. Endoscopic ultrasound (EUS) showed a cystic mass located along the greater curvature with no mucosal abnormality or communication with the gastric lumen. Magnetic resonance imaging performed for more characterization showed a cystic mass between the stomach and spleen with a high signal intensity on T2-weighted fat-saturated magnetic resonance images (Fig. 1) and peripheral enhancement after gadolinium injection (Fig. 2). At this stage, the differential diagnoses included hydatid cyst, mesenteric cyst, and pancreatic pseudocyst.

**Fig. 3:**
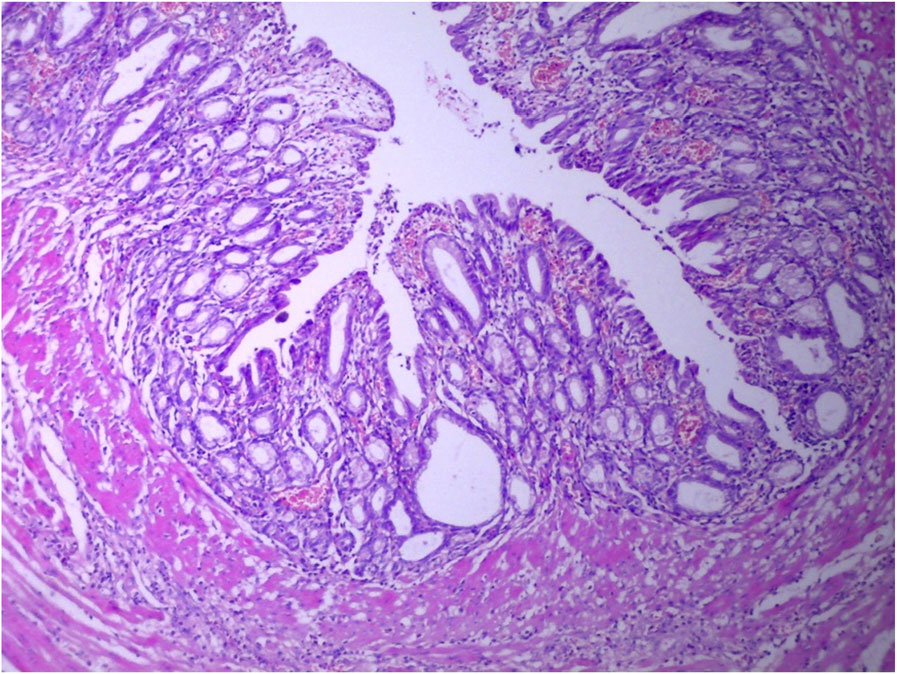
The cystic mass was lined by gastric epithelium with pyloric glands, and bundles of smooth muscle can be seen in the wall

**Fig. 4:**
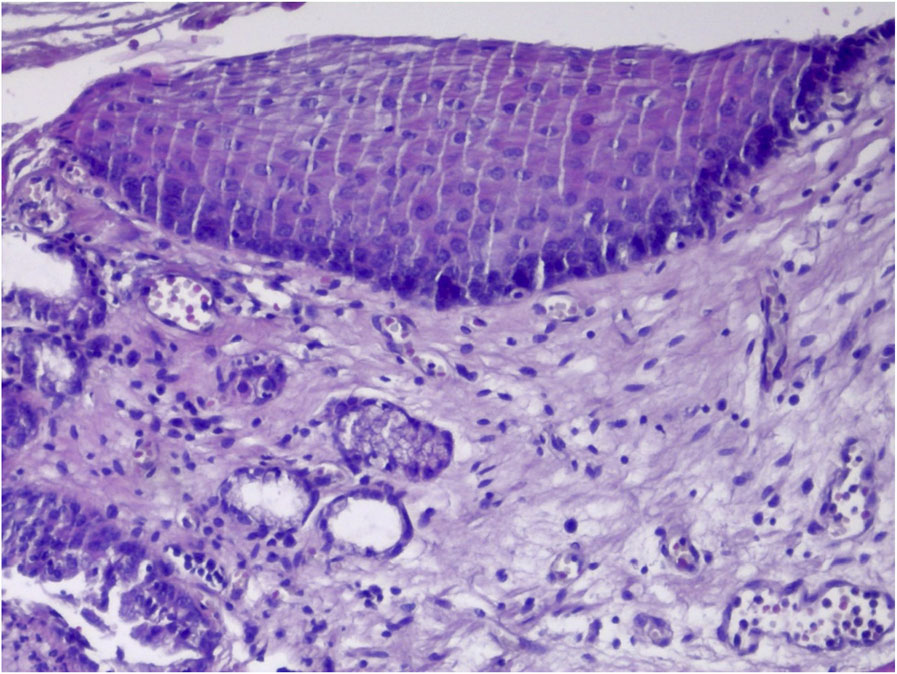
Squamous epithelium

**Fig. 5:**
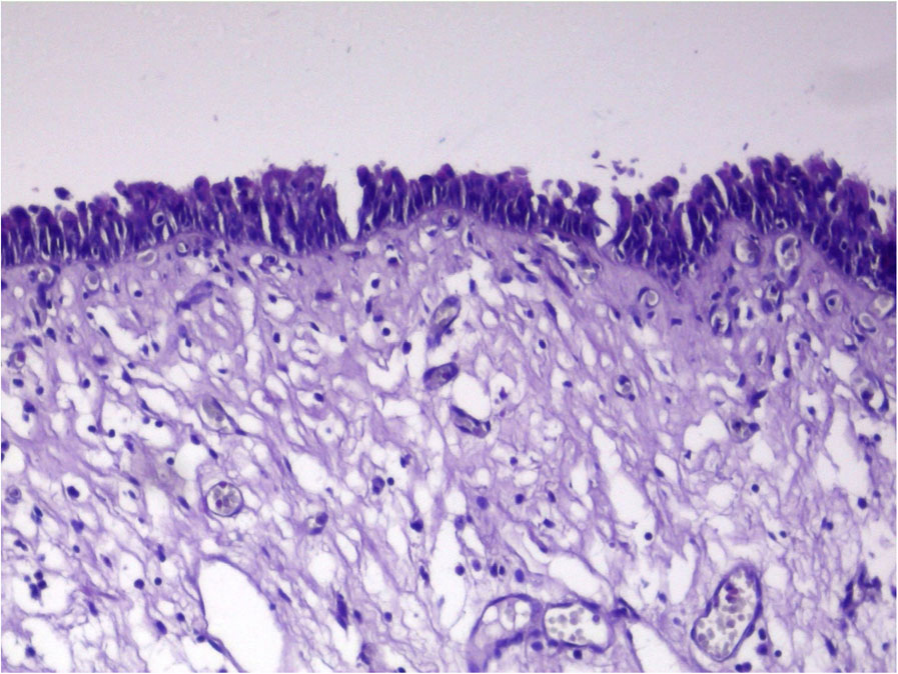
Respiratory pseudostratified epithelium

**Fig. 6:**
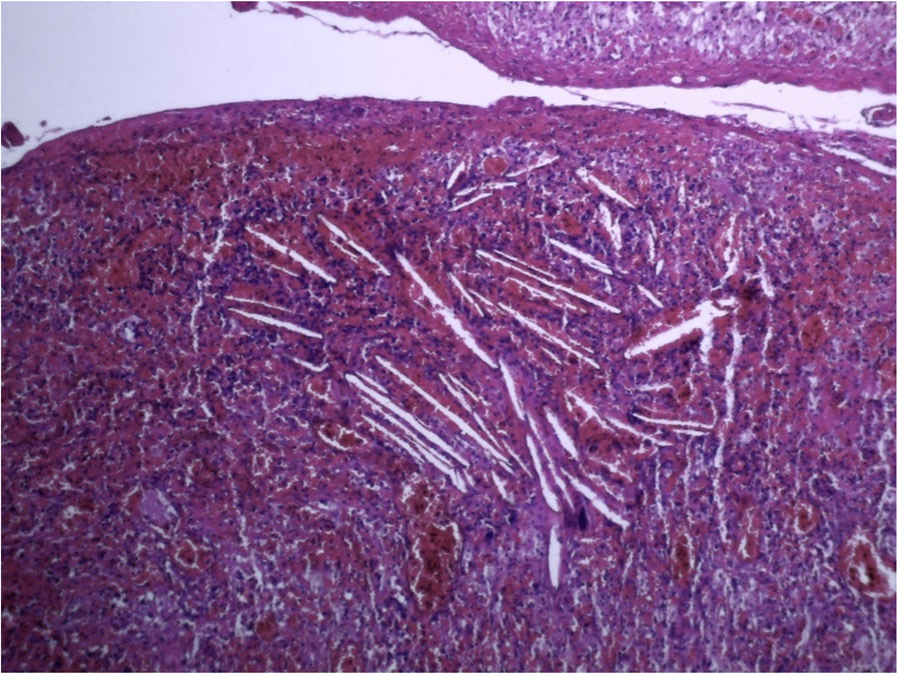
Area of ulceration and inflammation

The patient underwent exploratory laparotomy with complete cyst resection and splenectomy. At gross examination, the cystic mass, measuring 8 × 5.5 × 4 cm, was well-circumscribed, unilocular, and filled with a mucoid yellowish fluid. Histologically, the cystic mass was lined by gastric epithelium with pyloric glands (Fig. 3), transitioning focally into squamous epithelium (Fig. 4) and in some areas into pseudostratified columnar ciliated epithelium (Fig. 5). A small part of the mucosa-like wall had ulceration accompanied by inflammation (Fig. 6). There was no evidence of cartilaginous tissue in the wall. There were also bundles of smooth muscle in the wall. This finding suggested a GDC .

The histological examination of splenectomy showed pronounced cord congestion with reactive follicular hyperplasia and increased deposition of hemosiderin. After 10 months, there were no signs of local complication, and the patient was successfully weaned from steroids and had complete resolution of hemolytic anemia with negative direct antiglobulin tests.

## Discussion

In this report, we present a rare association of a hemolytic autoimmune anemia and an asymptomatic GDC in a 28-year-old man. This association has not been reported in the literature so far. We also describe a unique histological morphology of this cyst, which was lined by three different types of epithelium. Duplications of the alimentary tract are rare and occur in 1 in 4500 births [4]. Gastric duplications are infrequent and constitute 8% of all duplications. Duplication cysts of the ileum are usually located on the mesenteric border, whereas the usual location for GDCs is along the greater curvature [5], as in our patient. It is believed that they are congenital developmental alterations within the gastrointestinal tract. Many theories exist for the development of these lesions, including a persistent embryological diverticulum, aberrant recanalization of the alimentary tract, partial twinning, and *in utero* ischemic events [6]. The majority of cases are diagnosed in the pediatric population within the first 3 months of life and rarely in patients over 12 years of age [7].

There are two types of duplication: tubular when the lumen is contiguous with stomach lumen and cystic when the lumen is not contiguous with stomach lumen [8]. More than 80% of gastric duplications are cystic and do not communicate with the stomach lumen. The epithelial lining of duplication may be histologically similar to the segment to which the cyst is topographically related, or it may include, in a minority of cases, lining from another segment of the alimentary tract or cellular components that do not resemble those naturally occurring in the gastrointestinal tract, such as ciliated and respiratory-type epithelium and cartilage.

Our patient exhibited a unique histological finding: The lining epithelium was composed of both squamous and respiratory mucosa with the gastric epithelium, which has not been reported so far in the same cyst. The cyst lining may undergo erosions, ulceration, and regenerative changes due to increased fluid pressure. These changes may lead to bleeding into the cyst or perforation into the peritoneal cavity. These cysts are usually asymptomatic and are diagnosed incidentally, as observed in the cases in this report. However, in some cases, they have no specific symptoms, such as epigastralgia, vomiting, anemia, or recurrent episodes of pancreatitis. They may also manifest as complications such as gastric outlet obstruction, gastric perforation, and carcinoma arising in the cyst [9].

Our patient’s case represents a very rare association between GDCs and hemolytic autoimmune anemia. Hemolytic anemia is commonly associated with lymphoproliferative neoplasms, drugs, connective tissue diseases, and infections and are very rarely reported in association with ovarian dermoid cysts and mesenteric dermoid cysts.

There is only one reported case of a rare association between an enteric duplication cyst and autoimmune hemolytic anemia. In that case, there was an adenocarcinoma arising in the cyst, which refers to a paraneoplastic autoimmune hemolytic anemia [10]. In our patient, the microscopic study of the resected cyst did not show malignancy. Furthermore, because we performed a splenectomy at the same time and the hemolytic anemia resolution was obtained after surgery, we cannot give a definite conclusion on the underlying etiology. However, there is a strong possibility of association between the cyst and anemia. This case presented a real diagnostic conundrum. The recognition of this association requires further studies and more reported cases.

Although most of the diagnoses are made postoperatively, several imaging modalities have provided some informative findings. CT and EUS are very useful in identifying GDCs [9]. Classically, contrast-enhanced CT typically demonstrates GDC as a thick-walled cystic lesion with enhancement of the inner lining [11]. Calcification is occasionally observed.

EUS is useful in distinguishing between the intramural and extramural lesions of the stomach. Compared with CT, magnetic resonance imaging can provide additional information about cyst content. However, the nature of the fluid in the GDC is different in each case according to bleeding, chronic inflammation, or infection. Treatment is complete excision of the cyst to avoid the risk of possible complications such as perforation, hemorrhage, and the risk of neoplastic transformation [12, 13].

## Conclusions

A GDC is a rare congenital anomaly that is difficult to diagnose before complete resection. It should be considered in the differential diagnosis of abdominal cystic lesions. Its association with hemolytic autoimmune anemia raises the possibility of a morbid association and requires more reported cases.

## Abbreviations

Cr: Creatinine
CRP: C-reactive protein
CT: Computed tomography
GDC: Gastric duplication cyst
EUS: Endoscopic ultrasound
Hb: Hemoglobin
Hct: Hematocrit
MCV: Mean corpuscular volume
Plt: Platelets
RBC: Red blood cells
RC: Reticulocyte count
TP: Total protein
WBC: White blood cells
